# Vector outcomes after SMILE pro with the VISUMAX 800 for high versus moderate-to-low astigmatism: a contralateral eye comparison

**DOI:** 10.3389/fmed.2026.1859491

**Published:** 2026-06-03

**Authors:** Yuchen Mei, Yawen Hao, Xuanyu Yang, Aoran Zhang, Ziyuan Tian, Wentian Zhou, Hui Zhang

**Affiliations:** 1 Nanchang Bright Eye Hospital, Nanchang, Jiangxi, China; 2 School of Ophthalmology and Optometry, Jiangxi Medical College, Nanchang University, Nanchang, Jiangxi, China; 3 School of Queen Mary, Jiangxi Medical College, Nanchang University, Nanchang, Jiangxi, China; 4 The Second Clinical Medical College, Jiangxi Medical College, Nanchang University, Nanchang, Jiangxi, China

**Keywords:** aberration, Alpins vector analysis, high astigmatism, small incision lenticule extraction, SMILE Pro, VisuMax 800

## Abstract

**Purpose:**

To evaluate vector outcomes and higher-order aberrations (HOAs) in eyes with high astigmatism (HA) and their contralateral eyes with moderate-to-low astigmatism (MLA) after small-incision lenticule extraction (SMILE Pro) with automated cyclotorsion compensation.

**Methods:**

This single-center retrospective study included 30 patients (60 eyes) who underwent bilateral SMILE Pro using the VISUMAX 800 platform with OcuLign iris registration. The eyes were classified as HA [> 2.00 diopters (D)] or MLA (≤ 2.00 D) according to their preoperative cylinder magnitude. Visual acuity, refractive outcomes, Alpins vector parameters, intraoperative decentration, and HOAs were compared at 6 months after surgery.

**Results:**

The mean preoperative manifest cylinder was 2.38 ± 0.25 D in HA eyes and 1.41 ± 0.49 D in MLA eyes. At 6 months, uncorrected distance visual acuity was comparable between eye groups (*p* = 0.40). The correction index (0.95 ± 0.10 vs. 0.96 ± 0.12; *p* = 0.52), absolute angle of error (2.42 ± 2.49° vs. 1.99 ± 2.34°; *p* = 0.46), and index of success were similar between HA and MLA eyes. Residual cylinder and difference vector were nominally higher in HA eyes in the unadjusted analyses, but the paired differences were small (both 0.13 D) and did not remain statistically significant after false discovery rate correction. Horizontal intraoperative decentration and trefoil also showed nominal unadjusted differences that did not remain significant after FDR correction. Postoperative total HOAs, spherical aberration, and coma were comparable between groups.

**Conclusion:**

In this small retrospective contralateral-eye cohort, SMILE Pro with OcuLign-assisted cyclotorsion compensation showed favorable 6-month visual, refractive, and vector outcomes in eyes with astigmatism slightly above 2.00 D. These findings should be interpreted cautiously given the modest sample size, narrow HA range, and absence of a non-inferiority design.

## Introduction

Small-incision lenticule extraction (SMILE) has become a mainstream refractive procedure because its flapless design and use of a small incision may better preserve corneal biomechanical integrity (1, 2). However, astigmatic correction is more sensitive to alignment error than spherical correction because outcomes depend on accurate refractive planning, as well as precise axis alignment and centration. This issue is particularly relevant for eyes with high astigmatism (HA) [> 2.00 diopters (D)] as small alignment errors can lead to an increased angle of error (AE), higher difference vector (DV), and greater residual astigmatism. Among alignment-related factors, cyclotorsional rotation is particularly important because it directly changes the intended astigmatic treatment axis.

Cyclotorsion occurs when patients move from an upright sitting position to a supine position and can induce axis misalignment (3). In the VISUMAX 500 era, compensation relied largely on manual limbal marking, which may introduce errors of several degrees under routine clinical conditions (4). Even an axis misalignment of approximately 3° can reduce effective astigmatic correction by approximately 10%, and this effect becomes more clinically relevant as the number of preoperative cylinders increases (5). In addition, decentration-related asymmetric higher-order aberrations (HOAs), particularly coma, may further compromise visual quality in highly astigmatic eyes (6, 7).

The VISUMAX 800 platform incorporates OcuLign for iris registration-based cyclotorsion detection and axis compensation, together with CentraLign for centration assistance (8). These advances, which address axis misalignment and decentration, are expected to improve astigmatic correction accuracy and reduce undercorrection in eyes with HA.

Current research about the VISUMAX 800 has mainly emphasized its overall safety, efficacy, and refractive predictability, whereas dedicated evaluations of eyes with HA remain limited. More recently, Yang et al. reported that, in a Chinese cohort with myopia and low-to-moderate astigmatism, SMILE Pro using the VISUMAX 800 was associated with shorter lenticule creation time, lower optical zone decentration, lower postoperative vertical coma, and more favorable astigmatic vector outcomes than conventional SMILE using the VISUMAX 500 (9). Moreover, most available studies have focused on overall refractive outcomes or platform-level comparisons rather than contralateral-eye analyses specifically evaluating whether vector accuracy is maintained in HA eyes relative to fellow MLA eyes. Few studies have used a contralateral eye design to simultaneously examine Alpins vector outcomes, intraoperative decentration, postoperative visual quality, and HOAs (10–15). Employing a contralateral-eye paired design can minimize participant-specific confounding factors and enable thorough assessments of the incremental value of automated cyclotorsion compensation.

Therefore, this single-center retrospective contralateral eye study compared eyes with HA that underwent SMILE Pro with OcuLign to their fellow eyes with moderate-to-low astigmatism (MLA). We aimed to evaluate whether favorable correction efficiency and axis alignment accuracy could be maintained in HA eyes after SMILE Pro with automated cyclotorsion compensation. We evaluated visual acuity, refractive predictability, Alpins vector parameters [target induced astigmatism (TIA), surgically induced astigmatism (SIA), correction index (CI), AE, DV, and index of success (IoS)], intraoperative decentration, and postoperative HOAs as indicators of optical quality (16).

## Patients and methods

### Study design and participants

This study adhered to the tenets of the Declaration of Helsinki and was approved by the institutional Ethics Committee (No. 202512–01). Because of the retrospective design and use of de-identified clinical data, the requirement for additional written informed consent for study participation was waived by the ethics committee. All participants had provided written informed consent for the surgical procedure and the use of anonymized clinical data.

This single-center retrospective contralateral eye paired study consecutively enrolled patients who underwent bilateral SMILE Pro surgery (VISUMAX 800, software version 1.2.4; Carl Zeiss Meditec) at a tertiary eye center between May and August 2025 and completed at least 6 months of follow-up. The eyes were classified according to the absolute preoperative manifest refractive cylinder value: eyes with a cylinder of > 2.00 D were assigned to the HA group, and the contralateral eyes with a cylinder of ≤ 2.00 D were assigned to the MLA group, forming a 1:1 paired-eye comparison. Participant selection and outcome assessments were performed according to the Keratorefractive Lenticule Extraction guidelines (17).

The inclusion criteria were bilateral SMILE Pro, one eye meeting the HA definition and the fellow eye meeting the MLA definition, preoperative myopic astigmatism (manifest sphere < 0 D), and completion of at least 6 months of follow-up.

The exclusion criteria were prior ocular surgery, keratoconus or forme fruste keratoconus, active ocular surface disease, pregnancy or lactation, systemic disease affecting corneal wound healing, and device-related contraindications.

### Preoperative evaluation

All patients underwent standardized preoperative examinations including uncorrected distance visual acuity (UDVA), corrected distance visual acuity (CDVA), manifest refraction, dilated fundus, and slit-lamp biomicroscopy. Visual acuity was recorded in decimal notation and converted to the logarithm of the minimum angle of resolution for analyses. Contact lenses were discontinued for at least 7 days before the examinations.

Corneal tomography and ocular aberrations were measured using a Pentacam HR system (software version 1.21r43; Oculus). HOAs were quantified as the root mean square (RMS) of Zernike coefficients over a 6.0-mm analysis diameter, including the total HOA RMS, spherical aberration (SA), coma, and trefoil.

### Surgical technique

All surgical procedures were performed by a single experienced surgeon. The preoperative iris reference images were imported into the VISUMAX 800 system. After docking, the OcuLign iris registration was activated to detect cyclotorsion and compensate for the intended treatment axis before laser scanning. When indicated, CentraLign was used for visual-axis-guided centration using the corneal vertex as a reference.

The target refraction was individualized according to patient age and visual needs. The treatment axis was aligned with the preoperative manifest cylinder axis, and sphere/cylinder corrections were planned according to the surgeon’s nomograms. SMILE Pro was performed using standard VISUMAX 800 settings with the patient-specific parameters adjusted according to corneal thickness, refractive error, and residual stromal bed requirements.

The femtosecond laser parameters were set according to the manufacturer’s recommendations and the surgeon’s routine nomogram. The laser energy was set at 125 nJ, with a cap thickness of 120 μm, cap diameter of 7.6 mm, and side-cut angle of 120°. The intended optical zone was individualized according to the refractive correction and corneal thickness. The laser sequence consisted of posterior lenticule surface creation, lenticule side cut, anterior cap surface creation, and cap side cut. After completion of laser scanning, the incision was opened with a blunt dissector, and the anterior and posterior lenticule planes were sequentially separated. The lenticule was then extracted through the small incision using microforceps, and the interface was inspected to confirm complete removal without visible residual lenticule fragments.

### Postoperative regimen and follow-up

The patients were instructed to wear an eye shield at night for 1 week. Postoperative topical therapy included levofloxacin 0.5%, a corticosteroid (fluorometholone or tobramycin-dexamethasone), and sodium hyaluronate administered 4 times daily for 1 week.

Follow-up visits were scheduled at 1 week and 1, 3, and 6 months after surgery. At each visit, the UDVA, CDVA, and manifest refraction [sphere, cylinder, and spherical equivalent (SE)] were recorded. Ocular aberrations were reassessed at 6 months.

### Outcomes and Alpins vector analysis

The primary outcomes at 6 months included residual refractive cylinder, SE, UDVA, CDVA, and the Alpins vector parameters TIA, SIA, DV, magnitude of error, AE, CI, IoS, and intraoperative decentration parameters. HOAs, including the total HOA RMS, SA, coma, and trefoil, were also evaluated at 6 months. Because the intended postoperative astigmatic target was 0 D in all eyes, the magnitude of DV was mathematically equivalent to the magnitude of postoperative residual refractive astigmatism.

The secondary outcomes included sphere, cylinder, SE, UDVA, and CDVA at 1 week and at 1, 3, and 6 months. SE was calculated as sphere + cylinder/2. The eyes were categorized as undercorrected (< 0.90), ideally corrected (0.90–1.10), or overcorrected (> 1.10) based on the CI. Alpins vector calculations were conducted using a custom Microsoft Excel (Microsoft Corp.) worksheet based on standard formulas.

Double-angle vector plots were generated for TIA, SIA, and DV to visualize individual-eye vectors, vector means, 95% confidence ellipses of the dataset, and 95% confidence ellipses of the centroid. Astigmatic axes were doubled to account for the 180° periodicity of astigmatism. CI was not plotted as a vector because it is a scalar ratio rather than a directional astigmatic vector.

### Statistical analyses

Statistical analyses were performed using SPSS version 29.0 (IBM Corp.). Continuous variables are presented as means ± standard deviations for clinical interpretability. Normality was assessed using the Shapiro–Wilk test for paired-eye differences, defined as HA minus MLA. Variables with normally distributed paired-eye differences were compared using paired-samples t tests, whereas variables with non-normally distributed paired-eye differences were compared using Wilcoxon signed-rank tests. Categorical variables were compared using the McNemar test or reported descriptively, as appropriate. Paired mean differences were calculated as HA minus MLA, and 95% confidence intervals (CIs) were calculated for key outcomes. Cohen’s dz was calculated as the mean paired difference divided by the standard deviation of the paired differences. To account for multiple comparisons among key outcomes and exploratory correlation analyses, false discovery rate (FDR)-adjusted *p*-values were calculated using the Benjamini–Hochberg method. The *p-*values shown in the main tables are unadjusted, whereas FDR-adjusted *p-*values are provided in Supplementary Tables S1–S4. Spearman correlation analyses were performed separately in MLA and HA eyes to assess the associations between DV and preoperative manifest cylinder magnitude, postoperative UDVA, and HOA parameters, as well as the associations between intraoperative decentration and postoperative coma and trefoil. A *post-hoc* power analysis was performed for key Alpins vector outcomes, including DV, CI, and absolute AE, using a two-sided paired-samples *t*-test framework with *α* = 0.05. All tests were two-sided, and *p* < 0.05 was considered statistically significant. Detailed normality assessments, paired mean differences with effect sizes, *post-hoc* power analysis, and exploratory correlation analyses are provided in Supplementary Tables S1–S4.

## Results

### Preoperative baseline characteristics

The 30 participants (60 eyes) had a mean age of 20.07 ± 3.52 years. The MLA and HA groups had comparable results for the preoperative manifest sphere, SE, CDVA, central corneal thickness, mesopic pupil diameter, and total HOAs (all *p* > 0.05), indicating good baseline comparability. As expected, the mean preoperative manifest refractive cylinder (absolute value) was significantly greater in the HA group than in the MLA group (2.38 ± 0.25 D vs. 1.41 ± 0.49 D, *p* < 0.001) (Table 1).

**Table 1:** Preoperative and surgical characteristics of contralateral eyes undergoing SMILE Pro With OcuLign.

Parameter	MLA eyes (*n* = 30) mean ± SD	HA eyes (*n* = 30) mean ± SD	*P*-value
Preoperative characteristics			
Manifest sphere (D)	−3.61 ± 1.06	−3.42 ± 1.37	0.47
Manifest cylinder (D)*	1.41 ± 0.49	2.38 ± 0.25	**<0.001**
Spherical equivalent (D)	−4.31 ± 1.06	−4.65 ± 1.24	0.17
CDVA (logMAR)	−0.11 ± 0.05	−0.10 ± 0.05	0.13
Central corneal thickness (μm)	565.57 ± 25.93	565.67 ± 25.59	0.93
Mesopic pupil diameter (mm)	7.02 ± 0.78	7.03 ± 0.81	0.66
Total HOA RMS (μm)	0.43 ± 0.13	0.43 ± 0.15	0.79
Surgical characteristics			
Optical zone (mm)	6.79 ± 0.12	6.78 ± 0.12	0.68
Attempted sphere (D)	−4.84 ± 1.50	−4.04 ± 1.67	**0.02**
Attempted cylinder (D)	−1.41 ± 0.49	−2.38 ± 0.25	**<0.001**
X-axis decentration (mm)	0.19 ± 0.17	0.11 ± 0.16	**0.04**
Y-axis decentration (mm)	0.07 ± 0.15	0.06 ± 0.13	0.96
Total decentration (mm)	0.26 ± 0.15	0.21 ± 0.12	0.08

Data are presented as mean ± standard deviation (SD). MLA, moderate-to-low astigmatism (absolute manifest cylinder ≤ 2.00 D); HA, high astigmatism (absolute manifest cylinder > 2.00 D); CDVA, corrected distance visual acuity; HOA, higher-order aberration; RMS, root mean square. *Manifest cylinder is presented as absolute magnitude. *p*-values were calculated using paired tests appropriate for the contralateral-eye design.

### Intraoperative parameters

The mean planned optical zone was similar between groups (6.79 ± 0.12 mm in MLA vs. 6.78 ± 0.12 mm in HA; *p* = 0.68). The attempted sphere was less myopic in HA eyes than in MLA eyes (−4.04 ± 1.67 D vs. − 4.84 ± 1.50 D; *p* = 0.02), whereas the greater attempted cylinder in HA eyes reflected the group definition. Horizontal intraoperative decentration was nominally smaller in HA eyes than in MLA eyes (0.11 ± 0.16 mm vs. 0.19 ± 0.17 mm; unadjusted *p* = 0.04). However, vertical decentration (0.06 ± 0.13 mm vs. 0.07 ± 0.15 mm; *p* = 0.96) and total decentration (0.21 ± 0.12 mm vs. 0.26 ± 0.15 mm; *p* = 0.08) did not differ significantly between groups (Table 1).

### Visual and refractive outcomes

**Figure 1:**
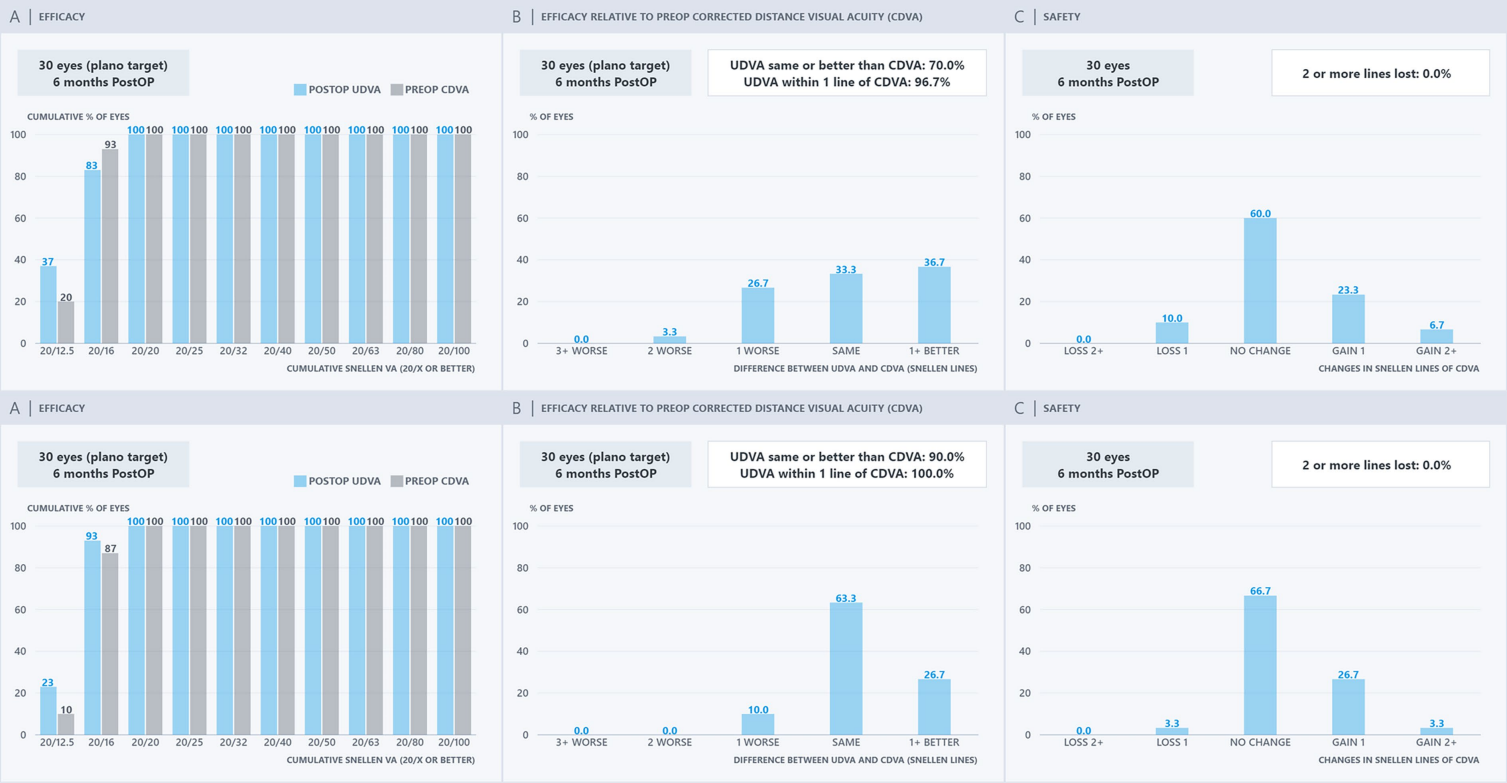
Visual efficacy and safety outcomes at 6 months after SMILE Pro. The graphs present a contralateral comparison between the moderate-to-low astigmatism group (upper row) and high astigmatism group (lower row). Standard labeling **(A–C)** is maintained for both rows to promote a direct vertical comparison. **(A)** Efficacy: cumulative distribution of postoperative UDVA compared with preoperative CDVA. **(B)** Efficacy: relative to the preoperative CDVA (change in Snellen lines). **(C)** Safety: changes in CDVA lines at 6 months. CDVA, corrected distance visual acuity; PostOP, postoperative; PreOP, preoperative; SMILE, small-incision lenticule extraction; UDVA, uncorrected distance visual acuity; VA, visual acuity.

At 1 week and 1, 3, and 6 months, the UDVA remained comparable between the groups (all *p* > 0.05). None of the eyes lost ≥ 2 lines of CDVA, indicating excellent safety (Figure 1).

**Figure 2:**
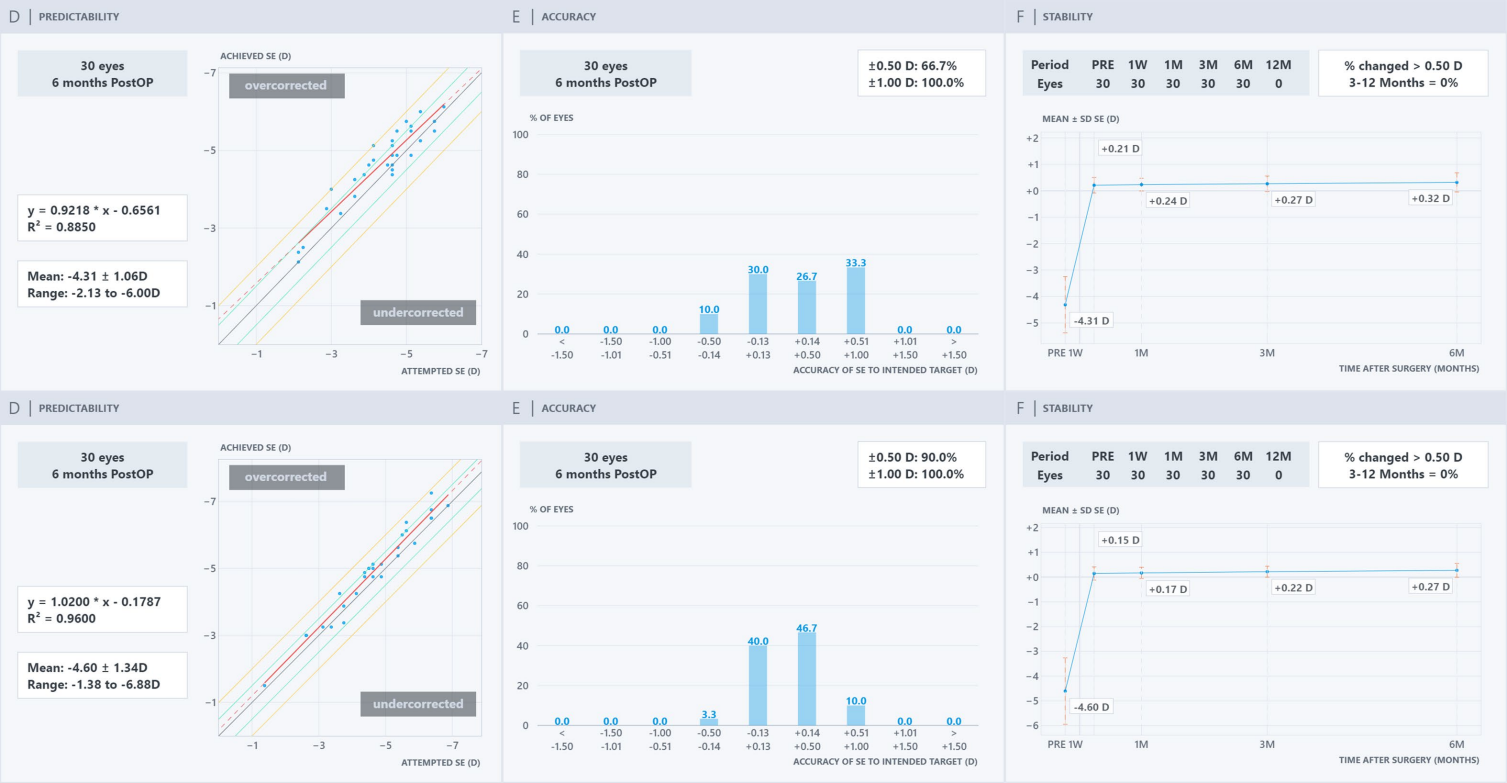
Predictability, accuracy, and stability of SE at 6 months. Comparisons are displayed for the MLA group (upper row) versus the HA group (lower row). **(A)** Predictability: attempted versus achieved SE, including the linear regression equation and coefficient of determination (*R*^2^). **(B)** Accuracy: histogram of the postoperative SE residual refractive error. **(C)** Stability: mean SE plotted over time from before surgery to 6 months after surgery. Standard graph labels **(A–C)** are used for both groups to allow side-by-side comparison. D, diopters; HA, high astigmatism; M, month; MLA, moderate-to-low astigmatism; PostOP, postoperative; PRE, preoperative; SE, spherical equivalent; W week.

At 6 months, the mean residual sphere was also similar between groups (0.41 ± 0.34 D vs. 0.42 ± 0.29 D; *p* = 0.83), as was the SE (0.32 ± 0.36 D vs. 0.27 ± 0.28 D; *p* = 0.54). Residual refractive cylinder magnitude was nominally higher in HA eyes than in MLA eyes across postoperative visits in unadjusted paired comparisons. At 6 months, residual cylinder was 0.31 ± 0.25 D in HA eyes and 0.18 ± 0.17 D in MLA eyes, with a mean paired difference of 0.13 D (95% CI, 0.03 to 0.23 D; unadjusted *p* = 0.02). However, this 6-month difference did not remain statistically significant after FDR correction (FDR-adjusted *p* = 0.11) (Table 2; Figure 2; Supplementary Table S2).

**Table 2:** Postoperative visual acuity and refractive outcomes at 1 week, 1 month, 3 months, and 6 months.

Outcome	Postoperative time	MLA eyes (*n* = 30) mean ± SD	HA eyes (*n* = 30) mean ± SD	*P-*value
UDVA (logMAR)				
	1 week	−0.07 ± 0.06	−0.07 ± 0.05	0.74
	1 month	−0.10 ± 0.05	−0.10 ± 0.06	0.87
	3 months	−0.14 ± 0.06	−0.13 ± 0.06	0.87
	6 months	−0.12 ± 0.07	−0.12 ± 0.06	0.40
SE (D)				
	1 week	0.34 ± 0.40	0.30 ± 0.35	0.55
	1 month	0.36 ± 0.37	0.27 ± 0.30	0.22
	3 months	0.35 ± 0.34	0.28 ± 0.25	0.37
	6 months	0.32 ± 0.36	0.27 ± 0.28	0.54
Sphere (D)				
	1 week	0.42 ± 0.40	0.45 ± 0.36	0.75
	1 month	0.43 ± 0.37	0.42 ± 0.30	0.82
	3 months	0.43 ± 0.31	0.43 ± 0.28	0.29
	6 months	0.41 ± 0.34	0.42 ± 0.29	0.83
Cylinder (D)				
	1 week	0.16 ± 0.15	0.31 ± 0.25	**0.01**
	1 month	0.15 ± 0.12	0.30 ± 0.25	**0.003**
	3 months	0.16 ± 0.14	0.30 ± 0.25	**0.01**
	6 months	0.18 ± 0.17	0.31 ± 0.25	**0.02**

Data are presented as mean ± standard deviation (SD). UDVA, uncorrected distance visual acuity (logMAR); SE, spherical equivalent, calculated as sphere + cylinder/2; D = diopters. Cylinder values are reported as absolute values (|cylinder|). *p-*values represent paired comparisons between contralateral eyes and were derived from paired-samples *t-*tests or Wilcoxon signed-rank tests, as appropriate. Unadjusted *p*-values < 0.05 are shown in bold. Two-sided *p*-values.

### Vector analysis

**Figure 3:**
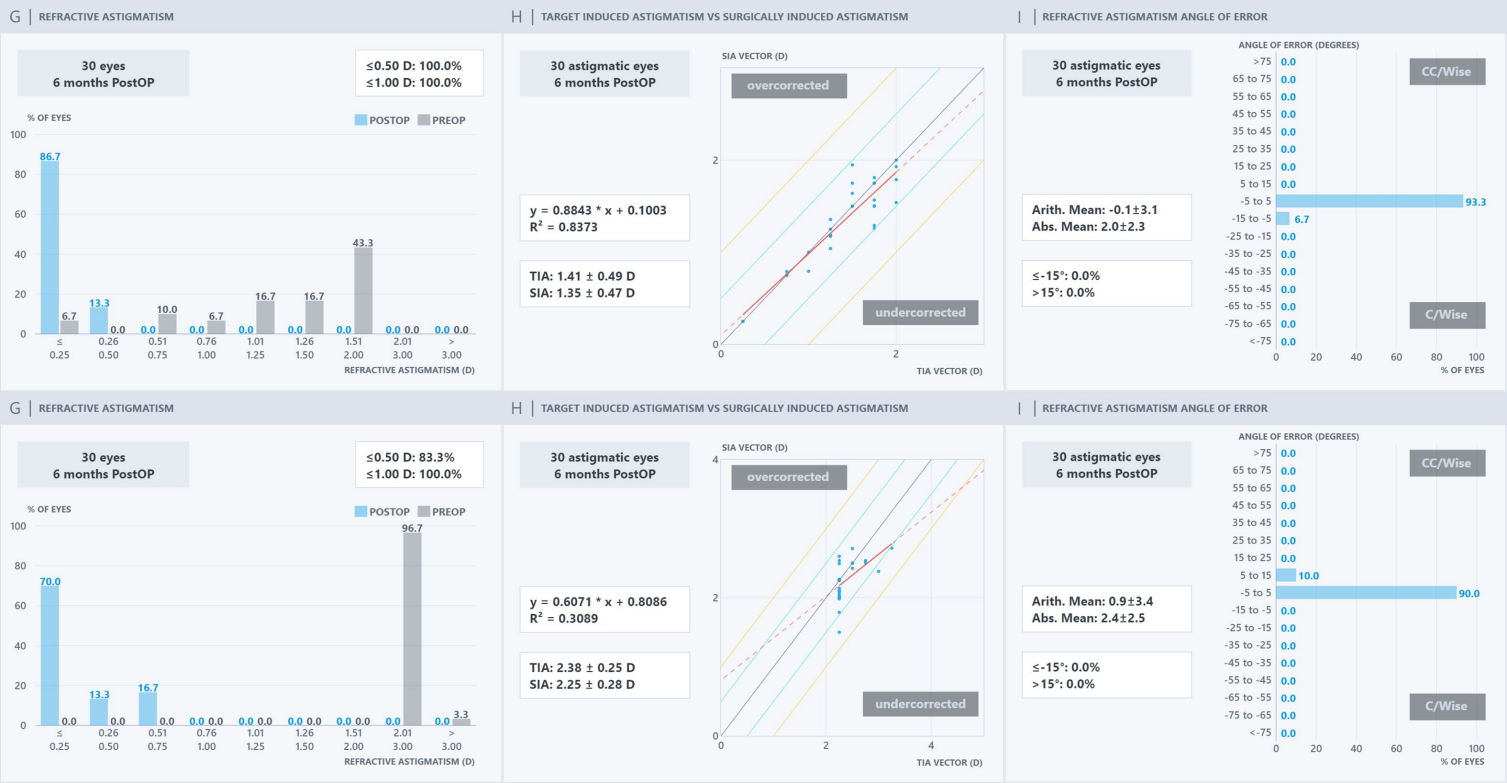
Alpins vector analysis of astigmatism correction at 6 months after SMILE Pro. The graphs present a contralateral comparison between the MLA group (upper row) and HA group (lower row). **(A)** Refractive astigmatism: distribution of preoperative versus postoperative astigmatism, with proportions of eyes achieving ≤ 0.50 D and ≤ 1.00 D indicated. **(B)** Vector predictability: TIA versus SIA linear regression analysis. Note the difference in R^2^ values, reflecting the narrower range of attempted astigmatism in the HA cohort than in the MLA cohort. The VISULYZE standard graph output uses automatic axis scaling; therefore, visual comparisons of TIA–SIA regression plots should be interpreted together with the quantitative vector parameters in Table 3 and Supplementary Table S2. **(C)** AE: distribution of the axis alignment error; arithmetic and absolute means AE are provided. Abs., absolute; AE, angle of error; Arith., arithmetic; C/Wise, clockwise; CC/Wise, counterclockwise; D, diopters; HA, high astigmatism; MLA, moderate-to-low astigmatism; PostOP, postoperative; PreOP, preoperative; SIA, surgically induced astigmatism; TIA, target induced astigmatism.

**Figure 4:**
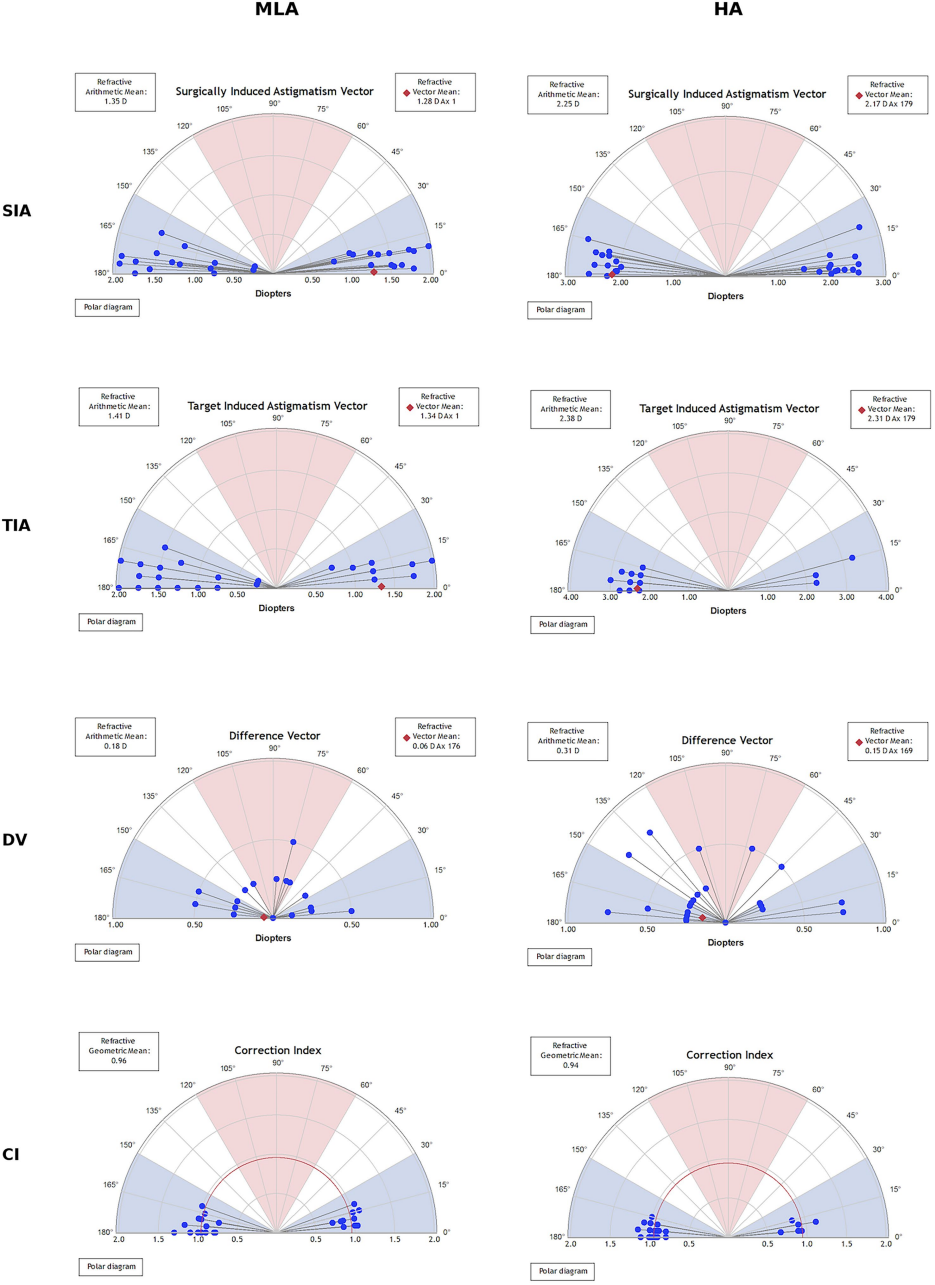
Alpins vector analysis of astigmatism correction at 6 months after SMILE Pro. The panels present a contralateral comparison between the MLA group (left column) and HA group (right column). Each row corresponds to a specific Alpins vector parameter: SIA, TIA, DV, and CI. Each polar plot displays individual eyes (blue points) and the vector mean (red diamond). Ax, axis; CI, correction index; D, diopters; DV, difference vector; HA, high astigmatism; MLA, moderate-to-low astigmatism; SIA, surgically induced astigmatism; SMILE, small-incision lenticule extraction; TIA, target induced astigmatism.

The Alpins vector analysis results are summarized in Table 3 and Figures 3, 4. As predicted, both the mean TIA and mean SIA were significantly greater in the HA eyes than in the contralateral MLA eyes (TIA: 2.38 ± 0.25 D vs. 1.41 ± 0.49 D, *p* < 0.001; SIA: 2.25 ± 0.28 D vs. 1.35 ± 0.47 D, *p* < 0.001). In both eye groups, the mean SIA was slightly lower than the mean TIA, and the CI was comparable (0.95 ± 0.10 vs. 0.96 ± 0.12, *p* = 0.52), indicating a similar mild tendency toward undercorrection.

**Table 3:** Alpins vector analysis of astigmatic correction at 6 months postoperatively (contralateral-eye comparison).

Parameter	MLA eyes (*n* = 30) mean ± SD	HA eyes (*n* = 30) mean ± SD	*P-*value
TIA	1.41 ± 0.49	2.38 ± 0.25	**<0.001**
SIA	1.35 ± 0.47	2.25 ± 0.28	**<0.001**
DV	0.18 ± 0.17	0.31 ± 0.25	**0.02**
ME	−0.06 ± 0.20	−0.12 ± 0.25	0.25
AE*	1.99 ± 2.34	2.42 ± 2.49	0.46
CI	0.96 ± 0.12	0.95 ± 0.10	0.52
IoS	0.12 ± 0.11	0.13 ± 0.10	0.73
Undercorrected eyes	9 (30.0%)	8 (26.7%)	
Ideally corrected eyes	19 (63.3%)	19 (63.3%)	
Overcorrected eyes	2 (6.7%)	3 (10.0%)	

Data are presented as mean ± standard deviation (SD) or n (%). TIA, target induced astigmatism; SIA, surgically induced astigmatism; DV, difference vector; ME, magnitude of error; AE, angle of error; CI, correction index (SIA/TIA); IoS, index of success (DV/TIA). AE is reported as the absolute value (degrees). *p-*values represent paired comparisons between contralateral eyes and were derived from paired-samples t tests or Wilcoxon signed-rank tests, as appropriate. Statistically significant *p-*values (< 0.05) are shown in bold. Because the target astigmatism was 0 D, the magnitude of DV is mathematically equivalent to the magnitude of postoperative residual astigmatism in this study. The distributions of undercorrection/ideal correction/overcorrection are reported descriptively. *AE is presented as absolute magnitude. Two-sided *p-*values.

**Figure 5:**
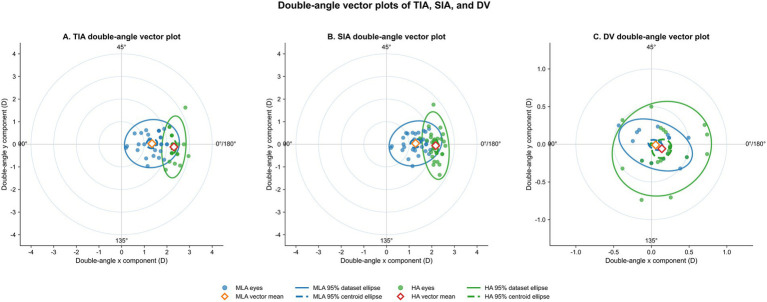
Double-angle vector plots of **(A)** TIA, **(B)** SIA, and **(C)** DV with vector means and 95% confidence ellipses. Double-angle vector plots showing the distributions of target induced astigmatism (TIA), surgically induced astigmatism (SIA), and difference vector (DV) in moderate-to-low astigmatism (MLA) and high astigmatism (HA) eyes. Astigmatic vectors were transformed into Cartesian coordinates using *x* = *M* cos(2*θ*) and *y* = *M* sin(2θ), where *M* represents the vector magnitude in diopters and θ represents the vector axis in degrees. Circles indicate individual eyes, and hollow diamonds indicate vector means. Solid ellipses represent 95% dataset ellipses, which describe the dispersion of individual vectors, whereas dashed ellipses represent 95% centroid ellipses, which describe the uncertainty around the vector mean. TIA and SIA are displayed using the same axis range to allow direct visual comparison, whereas DV is displayed on a smaller scale because of its lower magnitude. TIA, target induced astigmatism; SIA, surgically induced astigmatism; DV, difference vector; MLA, moderate-to-low astigmatism; HA, high astigmatism.

DV was nominally higher in HA eyes than in MLA eyes (0.31 ± 0.25 D vs. 0.18 ± 0.17 D; unadjusted *p* = 0.02), consistent with the slightly greater residual cylinder magnitude when the intended postoperative astigmatic target was 0 D. The mean paired difference in DV was 0.13 D (95% CI, 0.03 to 0.23 D), but this difference did not remain statistically significant after FDR correction (FDR-adjusted *p* = 0.11). ME, absolute AE, CI, and IoS were comparable between groups (all *p* > 0.05). Consistent with these findings, Figure 3 shows a marked postoperative refractive astigmatism reduction in both groups, with close agreement between the intended and achieved corrections in vector regression analysis. Figure 4 illustrates the distribution of vector parameters and CI patterns between the eye groups. To further visualize vector dispersion, Figure 5 presents double-angle vector plots for TIA, SIA, and DV with vector means and 95% confidence ellipses. The TIA and SIA plots showed broadly consistent vector distributions between intended and achieved astigmatic correction within each group, whereas the DV plot demonstrated small residual vector centroids in both groups. The proportion of ideally corrected eyes was identical in both groups (63.3%), with similar distributions of under- and overcorrections. *Post-hoc* power analysis showed achieved power values of 0.73 for DV, 0.10 for CI, and 0.11 for absolute AE (Supplementary Table S3).

### Aberration analysis

At 6 months, total HOA RMS, SA, horizontal coma, and vertical coma did not differ significantly between the eye groups (total HOA RMS: 0.62 ± 0.11 μm in MLA eyes vs. 0.60 ± 0.14 μm in HA eyes, *p* = 0.38; SA: 0.10 ± 0.07 μm vs. 0.08 ± 0.05 μm, *p* = 0.26; horizontal coma: −0.04 ± 0.11 μm vs. − 0.02 ± 0.10 μm, *p* = 0.30; vertical coma: 0.09 ± 0.10 μm vs. 0.10 ± 0.11 μm, *p* = 0.70). Trefoil was nominally higher in HA eyes (0.26 ± 0.04 μm vs. 0.24 ± 0.06 μm; unadjusted *p* = 0.04), with a small mean paired difference of 0.02 μm. This difference did not remain statistically significant after FDR correction (FDR-adjusted *p* = 0.12). Overall, no HOA parameter remained significantly different between groups after FDR correction (Table 4 and Supplementary Table S2).

**Table 4:** Higher-order aberrations at 6 months after SMILE Pro (contralateral-eye comparison).

Parameter	MLA eyes (*n* = 30) mean ± SD	HA eyes (*n* = 30) mean ± SD	*P-*value
HOA (μm)	0.62 ± 0.11	0.60 ± 0.14	0.38
SA (μm)	0.10 ± 0.07	0.08 ± 0.05	0.26
Horizontal coma (μm)	−0.04 ± 0.11	−0.02 ± 0.10	0.30
Vertical coma (μm)	0.09 ± 0.10	0.10 ± 0.11	0.70
Trefoil (μm)	0.24 ± 0.06	0.26 ± 0.04	**0.04**

Data are presented as mean ± standard deviation (SD). All aberrations are reported in micrometers (μm) for a 6.0-mm analysis diameter. HOA, higher-order aberrations (RMS); SA, spherical aberration. *p*-values represent paired comparisons between contralateral eyes and were derived from paired-samples *t*-tests or Wilcoxon signed-rank tests, as appropriate. Statistically significant *p*-values (< 0.05) are shown in bold. Two-sided *p*-values.

### Exploratory correlation analyses

**Figure 6:**
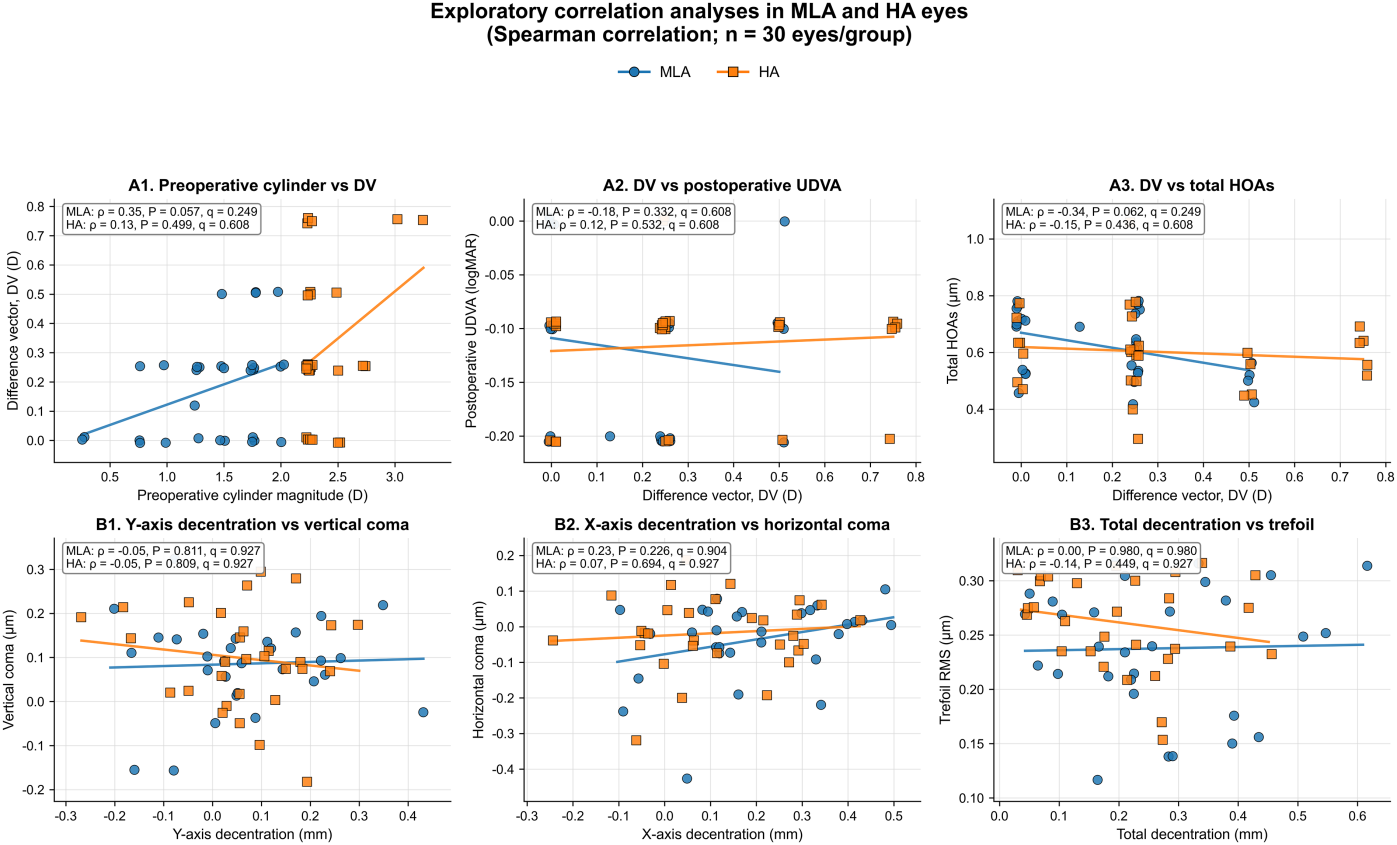
Exploratory correlation analyses in MLA and HA eyes. Scatter plots showing prespecified exploratory correlations in moderate-to-low astigmatism (MLA) and high astigmatism (HA) eyes. Panels show the associations between preoperative cylinder magnitude and the difference vector (DV) **(A1)**, DV and postoperative uncorrected distance visual acuity (UDVA, logMAR) **(A2)**, DV and total higher-order aberrations (HOAs) **(A3)**, Y-axis decentration and vertical coma **(B1)**, X-axis decentration and horizontal coma **(B2)**, and total decentration and trefoil RMS **(B3)**. Blue circles indicate MLA eyes, and orange squares indicate HA eyes. Spearman correlation coefficients (*ρ*), unadjusted *p-*values, and false discovery rate-adjusted *q* values are shown for each group. Scatter points were slightly jittered for visualization only; all correlation coefficients and *p-*values were calculated using the original, unjittered data. Linear fitted lines are shown for visual guidance only. DV, difference vector; HOAs, higher-order aberrations; RMS, root mean square.

Spearman correlation analyses were performed separately in MLA and HA eyes (Figure 6 and Supplementary Table S4). No significant correlation was observed between preoperative manifest cylinder magnitude and DV in either group (MLA: *ρ* = 0.35, unadjusted *p* = 0.06, FDR-adjusted *p* = 0.46; HA: ρ = 0.13, unadjusted *p* = 0.50, FDR-adjusted *p* = 0.73). DV was not significantly correlated with 6-month postoperative UDVA or total HOA RMS in either group (all FDR-adjusted *p* ≥ 0.46).

Intraoperative decentration parameters were not consistently associated with postoperative coma or trefoil. Horizontal decentration was not significantly correlated with horizontal coma in either group, and vertical decentration was not significantly correlated with vertical coma in either group. Total decentration was also not significantly correlated with trefoil in either group. A nominal association between total decentration and vertical coma was observed in HA eyes (ρ = 0.41, unadjusted *p* = 0.03), but this association did not remain significant after FDR correction (FDR-adjusted *p* = 0.46).

## Discussion

The contralateral eye-paired study design was implemented to minimize inter-individual confounding (corneal biomechanics, wound-healing response, and visual behavior). The effectiveness and vector precision of automated cyclotorsion compensation using the VISUMAX 800 platform (SMILE Pro) in eyes with HA was systematically assessed. Notably, the within-participant comparisons allowed direct evaluation of the marginal benefit for iris registration–based compensation under different astigmatic conditions.

The study findings suggest that using OcuLign (and CentraLign when needed) for eyes with HA (> 2.0 D) achieved postoperative UDVA comparable to that of their contralateral eyes with MLA and similar astigmatic correction efficiency indicated by the CI. Although residual astigmatism and trefoil showed nominal differences in the unadjusted analyses, these differences did not remain statistically significant after FDR correction and did not translate into clinically meaningful deterioration in visual outcomes during the 6-month follow-up period. Importantly, residual cylinder, DV, trefoil, and horizontal decentration showed only nominal differences in the unadjusted analyses, and these differences did not remain statistically significant after FDR correction.

Thus, these results suggest favorable early outcomes of SMILE Pro with automated cyclotorsion compensation in appropriately selected eyes with HA. In addition, the findings are broadly consistent with those of recent large cohort studies by Sekundo et al. and Ganesh et al., which demonstrated favorable safety and predictability of SMILE-based corrections, even in eyes with higher astigmatic burden (18, 19).

Prior studies about first-generation SMILE performed using the VISUMAX 500 platform have consistently reported a “magnitude-dependent attenuation” in astigmatic correction, with increasing undercorrection as the attempted cylinder value increases. Alio et al. suggested that undercorrection may increase by approximately 10–16% for each additional 1.00 D of astigmatism. Ivarsen et al. reported a marked decline in predictability for eyes with HA (> 3.00 D). Consequently, surgeons often rely on more aggressive or complex nomogram adjustments to compensate for this trend (20, 21). In contrast, the HA eyes in the present study did not demonstrate an apparent tendency toward undercorrection. The vector analysis demonstrated a CI of 0.95 for the HA eyes, which was comparable to that of the contralateral MLA eyes (0.96; *p* = 0.52). These findings suggest that, within this relatively narrow HA range slightly above 2.00 D, astigmatic correction efficiency was generally maintained with the VISUMAX 800 platform and iris registration-based cyclotorsion compensation. This observation is consistent with that of the report by Cung et al., indicating that OcuLign-assisted correction may mitigate the loss of astigmatic efficacy at greater magnitudes, and aligns with recent comparisons suggesting improved performance of VISUMAX 800 relative to VISUMAX 500 (8, 12, 22). Importantly, the within-participant design of this study further supports the interpretation that the undercorrection historically observed in HA may be a partial consequence of alignment-related factors (cyclotorsion and axis misregistration) rather than a strict limitation of the laser ablation/cutting capability. Therefore, these findings should not be directly extrapolated to eyes with very high astigmatism, particularly those exceeding 3.00 D.

Our findings may help inform procedure selection for patients with a higher astigmatic burden. Historically, toric implantable collamer lens (T-ICL) implantation has often been favored for astigmatism > 2.00 D because it preserves corneal tissue. However, T-ICL implantation has procedure-specific risks (cataract formation and intraocular pressure-related events) and its refractive stability is particularly sensitive to postoperative lens rotation (23, 24). Previous studies reported that a 10° rotation can result in an approximate 33% reduction in effective astigmatic correction, which may necessitate surgical repositioning (25, 26). In the present study, SMILE Pro with iris registration-based cyclotorsion compensation achieved a CI of approximately 0.95 in HA eyes, suggesting that a high level of astigmatic correction can be achieved without an intraocular implant. From a mechanistic perspective, SMILE Pro ascertains the intended treatment axis intraoperatively and applies corrections through tissue removal, thereby avoiding the postoperative “rotation” concern inherent to toric phakic intraocular lenses. The present study did not directly compare SMILE Pro with T-ICL; therefore, no conclusion can be drawn regarding procedural superiority. Rather, these findings suggest that SMILE Pro with automated alignment assistance may be a feasible corneal refractive option for appropriately selected patients with astigmatism slightly above 2.00 D.

Accurate axis alignment and centration are important determinants of astigmatic and optical outcomes. In the present study, horizontal intraoperative decentration was nominally smaller in HA eyes than in MLA eyes in the unadjusted analysis (0.11 mm vs. 0.19 mm; unadjusted *p* = 0.04). However, this difference did not remain statistically significant after FDR correction, and vertical decentration and total decentration did not differ significantly between groups. Therefore, the horizontal decentration finding should be interpreted cautiously and should not be considered evidence that HA eyes had superior centration accuracy.

The exploratory correlation analyses also did not show consistent associations between intraoperative decentration parameters and postoperative coma or trefoil. Horizontal decentration was not significantly correlated with horizontal coma, vertical decentration was not significantly correlated with vertical coma, and total decentration was not significantly correlated with trefoil in either group. Although total decentration showed a nominal association with vertical coma in HA eyes, this association did not remain significant after FDR correction. These findings suggest that the small nominal trefoil difference observed in HA eyes cannot be directly attributed to decentration based on the present data.

Despite favorable primary outcomes, residual refractive cylinder and DV were nominally higher in HA eyes in the unadjusted analyses, but these differences were small in magnitude and did not remain statistically significant after FDR correction. Importantly, these differences are better interpreted in the context of the baseline astigmatic magnitude than as evidence of inadequate correction. As the HA eyes had a substantially larger mean preoperative cylinder than (24) MLA eyes (2.38 D vs. 1.41 D), similar correction efficiency (CI ≈ 0.95–0.96) would be expected to yield a larger absolute residual magnitude in HA eyes. In addition, given that the target astigmatism was 0 D, the DV magnitude is mathematically equivalent to the postoperative residual astigmatism magnitude. Moreover, DV was not significantly correlated with postoperative UDVA or total HOA RMS in either group, suggesting that the small residual vector difference did not translate into measurable visual acuity or HOA deterioration. Clinically, the mean residual cylinder level in the HA eyes remained low (0.31 D) and the postoperative UDVA was comparable between eye groups, suggesting limited clinical impact from these nominal unadjusted differences. Trefoil was also nominally higher in HA eyes in the unadjusted analysis, but the absolute difference was small and did not remain statistically significant after FDR correction. This result aligns with the concept that correcting higher astigmatism involves a more elliptical lenticule profile, which may introduce subtle asymmetry-related aberrations (trefoil) through non-centrally symmetric biomechanical responses, as discussed by Chow et al. (6) However, the absolute between group difference was small (0.02 μm) and concomitant increases in SA or coma were not observed, supporting a generally stable postoperative aberration profile with SMILE Pro.

Beyond refractive precision, the high scanning speed of the VISUMAX 800 platform (with laser scanning time typically < 10 s per eye) may also contribute to procedural stability, particularly for HA eyes. Brar et al. reported that faster scanning shortens the suction time compared with the VISUMAX 500 platform, potentially reducing the risk of suction loss (15). Given that prolonged fixation may increase the likelihood of cyclotorsion or fixation instability, especially in challenging cases, an ultrafast workflow may serve as an additional safeguard for maintaining alignment and treatment accuracy, as observed by Ganesh et al. (27).

Despite the encouraging findings, this study has some limitations. First, as highlighted by Asif et al., postoperative biomechanical stabilization and epithelial remodeling after SMILE are dynamic processes that may evolve over time. Therefore, the 6-month follow-up period in the present study was relatively short and long-term observation is warranted to confirm the stability of HA correction (28). Second, the retrospective single-center design and modest sample size limit generalizability of the results. The *post-hoc* power analysis showed moderate power for detecting the observed DV difference but limited power for detecting small differences in CI and absolute AE. Therefore, the absence of statistically significant differences in CI and AE should not be interpreted as definitive equivalence between HA and MLA eyes. Third, the HA group had a relatively narrow cylinder range, with a mean preoperative manifest cylinder of 2.38 ± 0.25 D; therefore, the findings mainly apply to eyes slightly above the 2.00-D threshold and should not be extrapolated to eyes with very high astigmatism. Fourth, the cohort was relatively young, which may reflect the local refractive surgery population during the study period; nevertheless, the findings may not fully generalize to older patients with different ocular surface conditions, healing responses, or visual demands. Future prospective studies with larger cohorts and extended follow-ups that incorporate patient-reported outcomes and functional visual endpoints (contrast sensitivity and validated glare/halo questionnaires) would further strengthen clinical extrapolation.

In conclusion, in this small retrospective contralateral-eye cohort, SMILE Pro using the VISUMAX 800 with OcuLign-assisted cyclotorsion compensation achieved favorable 6-month visual, refractive, and vector outcomes in eyes with astigmatism slightly above 2.00 D. HA eyes showed comparable CI, absolute AE, IoS, UDVA, and HOA profiles relative to fellow MLA eyes. Residual cylinder and DV were nominally higher in HA eyes in the unadjusted analyses, but the paired differences were small and did not remain significant after FDR correction. Larger prospective studies including eyes with broader and higher astigmatic ranges are needed to further validate these findings.

## Data Availability

The original contributions presented in the study are included in the article/Supplementary material, further inquiries can be directed to the corresponding author.
