# Supplementary material for: Vector outcomes after SMILE pro with the VISUMAX 800 for high versus moderate-to-low astigmatism: a contralateral eye comparison
Source: Front Med (Lausanne). 2026 Jun 3;13:1859491. doi: 10.3389/fmed.2026.1859491 (PMC13272052; doi:10.3389/fmed.2026.1859491)
Supplement: Supplementary file 1 [file Table_1.pdf]

Supplementary Table S1. Normality assessment of paired differences for all manuscript-related continuous outcomes

Outcome	Shapiro – Wilk W	P	Distribution
Preoperative characteristics			
Manifest sphere, D	0.97	0.49	Normal
Manifest cylinder, D	0.82	< 0.001	Non-normal
Spherical equivalent, D	0.98	0.74	Normal
CDVA （logMAR）	0.60	< 0.001	Non-normal
Central corneal thickness (μ m)	0.95	0.16	Normal
Mesopic pupil diameter (mm)	0.88	0.002	Non-normal
Total HOA RMS (μ m)	0.95	0.17	Normal
Surgical characteristics			
Optical zone (mm)	0.73	< 0.001	Non-normal
Attempted sphere (D)	0.97	0.65	Normal
Attempted cylinder (D)	0.82	< 0.001	Non-normal
Intraoperative decentration-X (mm)	0.99	0.97	Normal
Intraoperative decentration-Y (mm)	0.97	0.66	Normal
Total decentration (mm)	0.96	0.30	Normal
Postoperative Visual Acuity and Refractive Outcomes			
Sphere at 1 week （D）	0.96	0.32	Normal
Cylinder at 1 week （D）	0.84	< 0.001	Non-normal
SE at 1 week （D）	0.99	0.95	Normal
UDVA at 1 week （logMAR）	0.81	< 0.001	Non-normal
Sphere at 1 month （D）	0.95	0.16	Normal
Cylinder at 1 month （D）	0.86	< 0.001	Non-normal
SE at 1 month （D）	0.98	0.70	Normal
UDVA at 1 month （logMAR）	0.87	0.002	Non-normal
Sphere at 3 months （D）	0.92	0.03	Non-normal
Cylinder at 3 months （D）	0.88	0.004	Non-normal
SE at 3 months （D）	0.97	0.42	Normal
UDVA at 3 months （logMAR）	0.87	0.002	Non-normal
Sphere at 6 months （D）	0.95	0.20	Normal
Cylinder at 6 months （D）	0.88	0.003	Non-normal
SE at 6 months （D）	0.98	0.78	Normal
UDVA at 6 months （logMAR）	0.90	0.01	Non-normal
Higher-Order Aberrations at 6 Months			
HOA （μm）	0.97	0.42	Normal
SA (μm)	0.95	0.13	Normal
Horizontal coma (μm)	0.97	0.66	Normal
Vertical coma (μm)	0.95	0.13	Normal

Outcome	Shapiro – Wilk W	P	Distribution
Preoperative characteristics			
Trefoil (μm)	0.97	0.46	Normal
Alpins Vector Analysis of Astigmatic Correction at 6 Months			
TIA	0.82	< 0.001	Non-normal
SIA	0.91	0.02	Non-normal
DV	0.88	0.003	Non-normal
ME	0.90	0.01	Non-normal
Absolute AE	0.94	0.08	Normal
CI	0.96	0.24	Normal
IoS	0.97	0.59	Normal

Normality was assessed using the Shapiro–Wilk test for paired-eye differences. Paired difference was defined as HA minus MLA. Variables with normally distributed paired differences were analyzed using paired-samples t tests, whereas variables with non-normally distributed paired differences were analyzed using Wilcoxon signed-rank tests. Residual cylinder was analyzed as the absolute magnitude. Visual acuity values were converted to logMAR units for statistical analysis. X and Y decentration values are signed coordinates; total decentration is the vector magnitude. HA = high astigmatism; MLA = moderate-to-low astigmatism; DV = difference vector; CI = correction index; AE = angle of error; IoS = index of success; HOA = higher-order aberration; RMS = root mean square; SE = spherical equivalent; UDVA = uncorrected distance visual acuity; CDVA = corrected distance visual acuity; D = diopters.
