# Supplementary material for: Vector outcomes after SMILE pro with the VISUMAX 800 for high versus moderate-to-low astigmatism: a contralateral eye comparison
Source: Front Med (Lausanne). 2026 Jun 3;13:1859491. doi: 10.3389/fmed.2026.1859491 (PMC13272052; doi:10.3389/fmed.2026.1859491)
Supplement: Supplementary file 2 [file Table_2.pdf]

Supplementary Table S2. Paired mean differences, 95% confidence intervals, and effect sizes for key outcomes

Outcome	Mean paired difference	95% CI	Cohen' s dz	P	FDR-adjusted P
UDVA at 6 months (logMAR)	0.01	−0.03 to 0.04	0.05	0.40	0.60
Cylinder at 6 months (D)	0.13	0.03 to 0.23	0.48	0.02	0.11
DV	0.13	0.03 to 0.23	0.48	0.02	0.11
CI	−0.01	−0.06 to 0.031	−0.12	0.52	0.63
Absolute AE	0.43	−0.74 to 1.61	0.14	0.46	0.61
IoS	0.01	−0.04 to 0.06	0.06	0.73	0.79
Total HOA RMS (μm)	−0.02	−0.07 to 0.03	−0.16	0.38	0.60
Horizontal coma (μm)	0.02	-0.02 to 0.06	0.19	0.30	0.64
Vertical coma (μm)	0.01	-0.05 to 0.08	0.07	0.70	0.79
Trefoil (μm)	0.02	0.002 to 0.05	0.40	0.04	0.12
Intraoperative decentration-X (mm)	−0.08	−0.15 to −0.004	−0.39	0.04	0.12
Intraoperative decentration-Y (mm)	−0.002	−0.07 to 0.07	−0.01	0.96	0.96
Total decentration (mm)	−0.06	−0.12 to 0.007	−0.33	0.08	0.19

Values were calculated based on paired-eye differences between HA and MLA eyes. Mean paired difference was defined as HA minus MLA. The 95% confidence interval was calculated for the paired-eye difference. Cohen's dz was calculated as the mean paired difference divided by the standard deviation of the paired differences. Residual cylinder was analyzed as the absolute magnitude. X and Y decentration values are signed coordinates; total decentration is the vector magnitude. P values were derived from paired-samples t tests or Wilcoxon signed-rank tests according to the normality of paired differences. FDR-adjusted P values were calculated using the Benjamini–Hochberg method. HA = high astigmatism; MLA = moderate-to-low astigmatism; 95% CI = 95% confidence interval; UDVA = uncorrected distance visual acuity; DV = difference vector; AE = angle of error; IoS = index of success; HOA = higher-order aberration; RMS = root mean square; D = diopters.
