# Supplementary material for: Vector outcomes after SMILE pro with the VISUMAX 800 for high versus moderate-to-low astigmatism: a contralateral eye comparison
Source: Front Med (Lausanne). 2026 Jun 3;13:1859491. doi: 10.3389/fmed.2026.1859491 (PMC13272052; doi:10.3389/fmed.2026.1859491)
Supplement: Supplementary file 3 [file Table_3.pdf]

Supplementary Table S3. Post-hoc power analysis for key Alpins vector outcomes

| Outcome | n | Mean paired difference | SD of paired difference | Cohen’s dz | Achieved power |
|---|---|---|---|---|---|
| DV, D | 30 | 0.13 | 0.27 | 0.48 | 0.73 |
| CI | 30 | −0.01 | 0.12 | −0.12 | 0.10 |
| Absolute AE, ° | 30 | 0.43 | 3.14 | 0.14 | 0.11 |

Values were calculated based on paired-eye differences between HA and MLA eyes. Mean paired difference was defined as HA minus MLA. Cohen’s dz was calculated as the mean paired difference divided by the standard deviation of the paired differences. Achieved power was estimated using a two-sided paired-samples t-test framework with $\alpha = 0.05$ and $n = 30$ pairs. Power estimates should be interpreted as approximate, particularly for outcomes with non-normally distributed paired differences. DV = difference vector; CI = correction index; AE = angle of error; HA = high astigmatism; MLA = moderate-to-low astigmatism; D = diopters.
