# Supplementary material for: Vector outcomes after SMILE pro with the VISUMAX 800 for high versus moderate-to-low astigmatism: a contralateral eye comparison
Source: Front Med (Lausanne). 2026 Jun 3;13:1859491. doi: 10.3389/fmed.2026.1859491 (PMC13272052; doi:10.3389/fmed.2026.1859491)
Supplement: Supplementary file 4 [file Table_4.pdf]

Supplementary Table S4. Spearman correlation analyses among DV, preoperative cylinder magnitude, postoperative visual and optical-quality parameters, and intraoperative decentration

Group	Analysis	Spearman's ρ	P value	Figure	FDR-adjusted P value
MLA	Preoperative cylinder vs DV	0.35	0.06	Figure 6	0.46
HA	Preoperative cylinder vs DV	0.13	0.50	Figure 6	0.73
MLA	DV vs UDVA	-0.18	0.33	Figure 6	0.73
MLA	DV vs total HOAs	-0.34	0.06	Figure 6	0.46
HA	DV vs UDVA	0.12	0.53	Figure 6	0.73
HA	DV vs total HOAs	-0.15	0.44	Figure 6	0.73
MLA	X-axis decentration vs horizontal coma	0.23	0.23	Figure 6	0.73
MLA	Y-axis decentration vs vertical coma	-0.05	0.81	Figure 6	0.85
MLA	Total decentration vs Trefoil	0.00	0.98	Figure 6	0.98
HA	X-axis decentration vs horizontal coma	0.07	0.69	Figure 6	0.85
HA	Y-axis decentration vs vertical coma	-0.05	0.81	Figure 6	0.85
HA	Total decentration vs trefoil	-0.14	0.45	Figure 6	0.73
MLA	DV vs horizontal coma	0.21	0.26	Table only	0.73
MLA	DV vs vertical coma	-0.15	0.43	Table only	0.73
MLA	DV vs trefoil	-0.19	0.33	Table only	0.73
MLA	Total decentration vs horizontal coma	0.32	0.09	Table only	0.49
MLA	Total decentration vs vertical coma	0.05	0.81	Table only	0.85
HA	DV vs horizontal coma	0.14	0.47	Table only	0.73
HA	DV vs vertical coma	-0.22	0.25	Table only	0.73
HA	DV vs trefoil	-0.05	0.81	Table only	0.85
HA	Total decentration vs horizontal coma	-0.17	0.37	Table only	0.73
HA	Total decentration vs vertical coma	0.41	0.03	Table only	0.46

Spearman correlation analyses were performed separately in eyes with moderate-to-low astigmatism and high astigmatism. Preoperative manifest cylinder was analyzed as the absolute magnitude. The difference vector was correlated with preoperative manifest cylinder magnitude, 6-month postoperative UDVA, total higher-order aberration RMS, horizontal coma, vertical coma, and trefoil. Intraoperative horizontal, vertical, and total decentration were correlated with postoperative horizontal coma, vertical coma, and trefoil. Horizontal and vertical decentration values are signed coordinates, whereas total decentration represents the vector magnitude. P values are two-sided and unadjusted unless otherwise specified. FDR-adjusted P values were calculated using the Benjamini–Hochberg method to account for multiple correlation analyses. UDVA = uncorrected distance visual acuity; RMS = root mean square; FDR = false discovery rate.
